# Analysis of entropy production in a bi-convective magnetized and radiative hybrid nanofluid flow using temperature-sensitive base fluid (water) properties

**DOI:** 10.1038/s41598-022-16059-9

**Published:** 2022-07-12

**Authors:** Tapas Barman, S. Roy, Ali J. Chamkha

**Affiliations:** 1 Mathematics Department, IIT Madras, Chennai, 600036 India; 2 Faculty of Engineering, Kuwait College of Science and Technology, 35004 Doha District, Kuwait

**Keywords:** Nanoscale materials, Theory and computation, Mechanical engineering, Fluid dynamics

## Abstract

The heat transport characteristics, flow features, and entropy-production of bi-convection buoyancy induced, radiation-assisted hydro-magnetic hybrid nanofluid flow with thermal sink/source effects are inspected in this study. The physical characteristics of hybrid nanofluids (water-hosted) are inherited from the base liquid (water) and none has considered the physical characteristics of base liquid (water) in the study of temperature-sensorial hybrid nanofluid investigations, though the water physical characteristics are not constants in temperature variations. So, the temperature-sensorial attributes of base liquid (water) are taken into account for this hybrid nanofluid ($Cu+{Al}_{2}{O}_{3}+\text{water}$) flow analysis. The mathematical forms of the flow configuration (i.e., the set of coupled, nonlinear PDE form of governing equations) are solved by utilizing the subsequent tasks: (i) congenial transformation; (ii) quasilinearization; (iii) methods of finite differences to form block matrix system, and (iv) Varga’s iterative algorithm. The preciseness of the whole numerical procedure is ensured by restricting the computation to follow strict convergence conditions. Finally, the numerically extracted results representing the impacts of various salient parameters on different profiles ($F, G, H$), gradients, and entropy production are exhibited in physical figures for better perception. A few noticeable results are highlighted as: velocity graph shows contrast behaviour under assisting and opposing buoyancy; temperature ($G(\xi ,\eta )$) is dropping for heightening heat source ($Q$) surface friction remarkably declines with the outlying magnetic field ($St$); thermal transport confronts drastic abatement under radiation (${R}_{1}$), and $St$; the characteristics Reynolds and Brinkman numbers promote entropy. Furthermore, the bounding surface acts as a strong source of ${S}_{G}$-production. Summarizations are listed at the end to quantify percentage variations.

## Introduction

The study of boundary layer (BL) flow along an inclined surface is enriched with real-life engineering applications like material processing, making glass fibres, solar energy systems, etc. Not only for its’ wide application, but this particular geometric flow has also been a challenge to interested researchers to enumerate the flow phenomenon and heat-mass transport characteristics. In early studies, pioneer researchers^1–3^ studied this geometry with different aspects of non-constant wall temperature, different inclination angle, different Prandtl numbers, etc. An experimental study of naturally convective flow for an inclined plate is presented by Al-Arabi and others^4^. Lewandowski^5^ studied naturally convective flow along an inclined plate with a new approach. Jayaraj^6^ inspected the thermophoretic effects on the flow for inclined plates. Later, a naturally convective flow was investigated for particulate suspension for inclined (isothermal) and vertical permeable plates by Ramadan and Chamkha^7,8^. A study of radiative MHD flow with variable porosity along an inclined plate was carried out by Chamkha and others^9^. Alam et al.^10^ reported the MHD effect in combination with variable suction, radiation effect on a permeable flow over inclined plate (semi-infinite). The study of boundary layer (BL) flow for vertical and inclined surfaces is further continued by several researchers^11–16^ considering different fluids (nanofluids, micro-polar fluids, etc.), and salient influencing factors like radiation, thermal injection/suction, outlying magnetic field, etc. An outlying magnetic field situating near an electrically conducting BL flow has numerous industrial engineering applications^17–22^. For example, in material processing, MHD effect may be used to get desired material structure^23^. Furthermore, the above-mentioned impactful factors in hybrid nanofluid flow encountered numerous applications in solar power technology, industrial areas, nuclear engineering, etc.,^24,25^. Recent studies^26,27^ showed that hybrid nanofluid is the most sensitive one in thermal transport means than ordinary fluid and nanofluids. Many studies on radiation, thermal source/sink, and MHD effects on hybrid nanofluid flow are available in current literature and a few of them are referred in the following texts^28–33^. Moreover, in any thermo-dynamical system, the engineering efficiency of the system degrades due to irreversible heat loss. The enumeration of irreversibility i.e., entropy generation (EG) of a system may help to minimize the irreversible heat loss. The application and importance of the EG-study of radiative MHD hybrid nanofluid flow affected by thermal sink/sources from biomedical point of view is explored by P.B.A. Reddy^34^. Researchers^35^ have found significant contributions of EG analysis in the studies of brain dynamics. Few more remarkable studies on this context are added as references^36–40^.

It is a common practice to use water as a base liquid but water and water-hosted nanofluids are temperature-sensitive. Besides the thermos physical nanofluid characteristics are inherited from the hosted liquid, those properties are enhanced, advanced and empowered by the properties (thermos-physical) of emerging nanoparticles. But in recent studies, it is observed that base fluid properties have been ignored in temperature-sensitive nanofluid flow investigations. So, authors have investigated the temperature-sensorial characteristics (thermos-physical) of hybrid nanofluids in the light of temperature-sensorial water characteristics. That is, this study is taking account the temperature-sensorial properties of water into the model^41^ for thermal relations utilizing empirical data^42^ and used them to analyze the hybrid nano-liquid flow. Furthermore, the equations presenting the physical meaning of the considered physical system in mathematical form are solved using the following complicated numerical tasks^43,44^: (i) congenial transformation; (ii) quasilinearization; (iii) methods of finite-differences to form block matrix system, and (iv) Varga’s iterative algorithm. The preciseness of the numerical approach is preserved by employing a strict convergence criterion.

## Governing equations

From Table 1, ${\mu }_{f}$ and ${(Pr)}_{f}$ can be approximated at different temperatures as^41,42,44^

1 $${\mu }_{f}\left(T\right)=\frac{1}{{a}_{1}+{a}_{2}T},$$

2 $${(Pr)}_{f}\left(T\right)=\frac{1}{{b}_{1}+{b}_{2}T},$$

where constant coefficients obtained from the curve fitting of thermos-physical data of water at various temperatures are ${b}_{1}, {b}_{2}, {c}_{1}$ and ${c}_{2}$ defined as:

**Table 1:** Water properties vs. temperatures^44,45^.

T (C)	ρ (g $\text{ c}{\text{m}}^{-3})$	${\text{C}}_{\text{p}}$ $(\text{J }{10}^{7} {\text{kg}}^{-1} {\text{K}}^{-1})$	k $(\text{erg }{10}^{5} {\text{cm}}^{-1} {\text{s}}^{-1} {\text{K}}^{-1}$)	$\upmu$ ($\text{g }{10}^{-2} {\text{cm}}^{-1} {\text{s}}^{-1}$)	Pr
0	1.00228	4.2176	0.5610	1.7930	13.4
10	0.99970	4.1921	0.5800	1.3070	9.45
20	0.99821	4.1818	0.5984	1.0060	7.03
30	0.99565	4.1784	0.6154	0.7977	5.12
40	0.99222	4.1785	0.6305	0.6532	4.32
50	0.98803	4.1806	0.6435	0.5470	3.55

$$\left(\begin{array}{c}{b}_{1}\\ {b}_{2}\\ {c}_{1}\\ {c}_{2}\end{array}\right)=\left(\begin{array}{c}53.41\\ 2.43\\ 0.068\\ 0.004\end{array}\right).$$

The hybrid nanofluid-base liquid correlations for various physical characteristics are given below^46^

3 $${\mu }_{hnf}\left(T,\phi \right)=\frac{{\mu }_{f}\left(T\right)}{\sqrt{{(1-\phi )}^{5}}}; \phi ={\phi }_{{s}_{1}}+{\phi }_{{s}_{2}},$$

4 $$\frac{{\rho }_{hnf}\left(T,\phi \right)}{{\rho }_{f}\left(T\right)}=\left(1-\phi \right)+ \frac{{\rho }_{{s}_{1}}}{{\rho }_{f}\left(T\right)}{\phi }_{{s}_{1}}+\frac{{\rho }_{{s}_{2}}}{{\rho }_{f}\left(T\right)}{\phi }_{{s}_{2}},$$

5 $$\frac{{(\rho \beta )}_{hnf}\left(T,\phi \right)}{{(\rho \beta )}_{f}\left(T\right)}=\left(1-\phi \right)+ \frac{{(\rho \beta )}_{{s}_{1}}}{{(\rho \beta )}_{f}\left(T\right)}{\phi }_{{s}_{1}}+\frac{{(\rho \beta )}_{{s}_{2}}}{{(\rho \beta )}_{f}\left(T\right)}{\phi }_{{s}_{2}},$$

6 $$\frac{{{(C}_{p}\rho )}_{hnf}\left(T,\phi \right)}{{{(C}_{p}\rho )}_{f}\left(T\right)}=\left(1-\phi \right)+ \frac{{{(C}_{p}\rho )}_{{s}_{1}}}{{{(C}_{p}\rho )}_{f}\left(T\right)}{\phi }_{{s}_{1}}+\frac{{{(C}_{p}\rho )}_{{s}_{2}}}{{{(C}_{p}\rho )}_{f}\left(T\right)}{\phi }_{{s}_{2}},$$

7 $$\frac{{\sigma }_{hnf}\left(T,\phi \right)}{{\sigma }_{f}\left(T\right)}=1+ \frac{3\phi \left({\Psi }_{1}-\phi {\sigma }_{f}\right)}{{\Psi }_{2}-\left({\Psi }_{1}-\phi {\sigma }_{f}\right)\phi } ; {\Psi }_{1}={\left(\sigma \phi \right)}_{{s}_{1}}+{\left(\sigma \phi \right)}_{{s}_{2}} ; {\Psi }_{2}={\Psi }_{1}+2\phi {\sigma }_{f},$$

8 $$\frac{{k}_{hnf}\left(T,\phi \right)}{{k}_{f}\left(T\right)}= \frac{\left(sf-1\right){k}_{f}\left(T\right)+\frac{{\Psi }_{3}}{\phi }-(sf-1)\left(\phi {k}_{f}\left(T\right)-{\Psi }_{3}\right)}{(sf-1){k}_{f}\left(T\right)+\phi {k}_{f}\left(T\right)+\left(\frac{{\Psi }_{3}}{\phi }-{\Psi }_{3}\right)} ; {\Psi }_{3}={\left(\phi k\right)}_{{s}_{1}}+{\left(\phi k\right)}_{{s}_{2}}.$$

Here $sf\left(=\frac{3}{\Omega }\right)$ stands for nanoparticles’ shape factor ($\Omega$ is the sphericity) (see Table 2) and the other terms $\phi , {\mu }_{hnf}, {\rho }_{hnf}, {\beta }_{hnf}, {\left({C}_{p}\right)}_{hnf}, {k}_{hnf}, {\sigma }_{hnf}, {\mu }_{f}, {\rho }_{f}, {\beta }_{f},{\left({C}_{p}\right)}_{f}, {k}_{f}, {\sigma }_{f}, {\phi }_{{s}_{1}}, {\rho }_{{s}_{1}},{\beta }_{{s}_{1}}, {\left({C}_{p}\right)}_{{s}_{1}} , {k}_{{s}_{1}}, {\sigma }_{{s}_{1}}, {\phi }_{{s}_{2}}, {\rho }_{{s}_{2}}, {\beta }_{{s}_{1}},{\left({C}_{p}\right)}_{{s}_{2}}, {k}_{{s}_{2}}, {\sigma }_{{s}_{2}}$ are all given in the Nomenclature.

**Table 2:** Nanoparticles’ shape and sphericity^47,48^.

Shape	Sphericity ($\Omega$)	Shape factor ($sf$)
Spherical	$1$	$3$
Bricks	$0.81$	$3.7$
Cylindrical	$0.62$	$4.9$
Platelets	$0.52$	$5.7$
Blade	$0.36$	$8.6$

Table 2 shows that the variation in ${\rho }_{f}, {\left({C}_{p}\right)}_{f}$ with respect to temperature is less than $1\%$. Combining this fact with the correlations (Eqs. 4–5) can be easily prove that the variation in ${\rho }_{hnf}, {\left({C}_{p}\right)}_{hnf}$ is also less than $1\%$. So, from practical point of view, these two physical quantities can be considered as constant (see Table 3).

**Table 3:** Nanoparticle properties^49,50^.

Properties	Copper	Alumina
${C}_{p} ({\text{Jkg}}^{-1} \; {\text{K}}^{-1})$	385	765
ρ ($\text{kg }\; {\text{m}}^{-3} )$	8933	3970
k (${\text{Wm}}^{-1}\; {\text{K}}^{-1}$)	400	40
β $\times {10}^{-5}\; {\text{K}}^{-1}$	1.67	0.85

**Figure 1:**
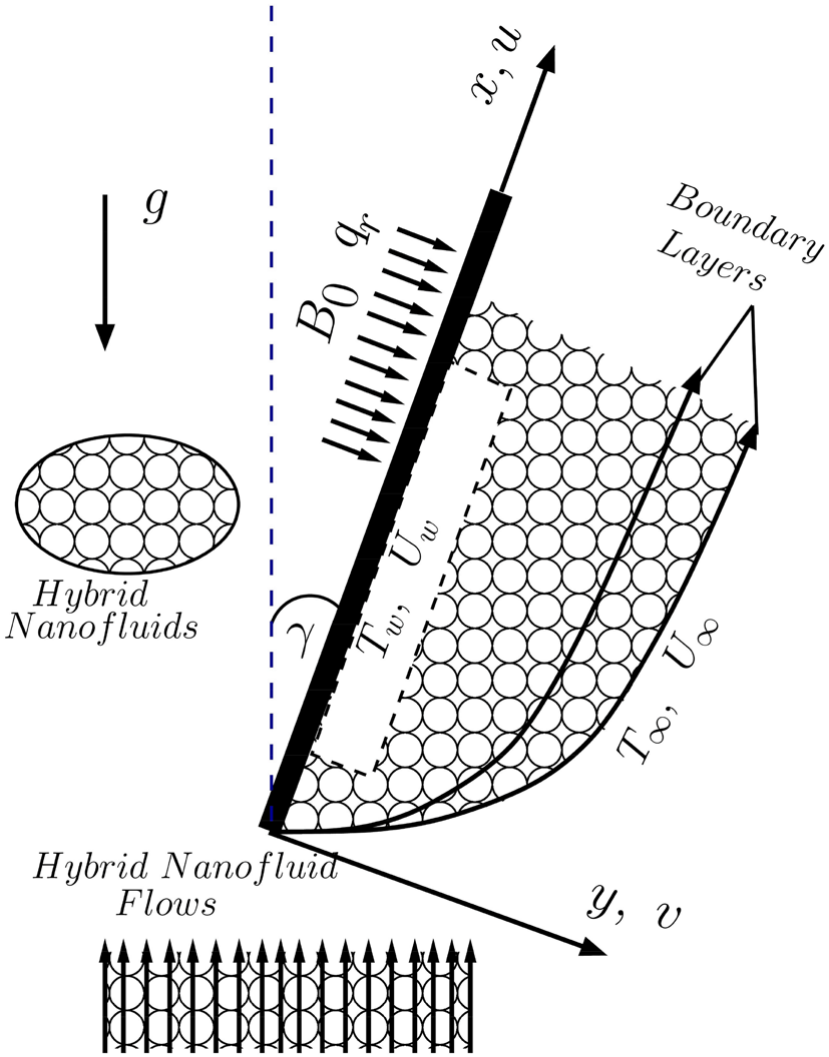
Flow geometry.

Consider a 2-D bi-convective (incompressible and steady) water-based hybrid-nanofluid flow for an arbitrarily inclined plate with vertical inclination $\gamma$ and let the axes $x$ and $y$ are along the surface and perpendicular to it, respectively (see Fig. 1). The convective variation in temperature from the wall to the ambient fluid is deemed moderate ($<{40 }$ °C) and an outer magnetic field normal to $x$-axis is applied under thermal sink/source and radiation effects. Using Oberbeck–Boussinesq approximation, the equations representing the physical characteristics of the flow become^10,51,52^

9 $$\overrightarrow{\nabla }.\overrightarrow{q}=0 ; \overrightarrow{q}\equiv \left(u,v\right),$$

10 $$\overrightarrow{q}.\overrightarrow{\nabla }u= \frac{1}{{\rho }_{hnf}}\frac{\partial }{\partial y}\left({\mu }_{hnf}\frac{\partial u}{\partial y}\right)+\frac{g{\left(\rho \beta \right)}_{hnf}\text{cos}\gamma }{{\rho }_{hnf}}\left(T-{T}_{\infty }\right)-\frac{{\sigma }_{hnf}{B}_{0}^{2}}{{\rho }_{hnf}}\left(u-{U}_{\infty }\right),$$

11 $$\overrightarrow{q}.\overrightarrow{\nabla }T= \frac{1}{{{(\rho C}_{p})}_{hnf}}\frac{\partial }{\partial y}\left({k}_{hnf}\frac{\partial T}{\partial y}\right)+\frac{{Q}_{0}}{{{(C}_{p}\rho )}_{hnf}}\left(T-{T}_{\infty }\right)-\frac{1}{{{(C}_{p}\rho )}_{hnf}}\frac{\partial {q}_{r}}{\partial y},$$

where ${\rho }_{s}=\frac{{\phi }_{{s}_{1}}{\rho }_{{s}_{1}}+{\phi }_{{s}_{2}}{\rho }_{{s}_{2}}}{{\phi }_{{s}_{1}}+{\phi }_{{s}_{2}}}; {{(C}_{p}\rho )}_{s}=\frac{{\phi }_{{s}_{1}}{{(C}_{p}\rho )}_{{s}_{1}}+{\phi }_{{s}_{1}}{{(C}_{p}\rho )}_{{s}_{2}}}{{\phi }_{{s}_{1}}+{\phi }_{{s}_{2}}}$” and ${q}_{r}= - \frac{4{\sigma }^{*}}{3{k}^{*}}\frac{\partial {T}^{4}}{\partial y}$. The non-linear term ${T}^{4}$ is approximated as $4{T}_{\infty }^{3}T-{3 T}_{\infty }^{4}$ (Roseland approximation) and hence finally $\frac{\partial {q}_{r}}{\partial y}$ becomes $-\frac{16 {\sigma }^{*} {T}_{\infty }^{3}}{3{k}^{*}}\frac{{\partial }^{2}T}{\partial {y}^{2}}$.

The constraints are given by:

12 $$u={U}_{w}; T= {T}_{w}; v= 0 ~ at ~ y=0,$$

13 $$u\to {U}_{\infty }; T\to {T}_{\infty }; ~ as ~ y\to \infty .$$

The following conversion variables

$$\xi =\frac{x}{L}; \eta =\sqrt{\left(\frac{U}{x{\nu }_{\infty }}\right)} y; U={U}_{w}+{U}_{\infty }; \epsilon =\frac{{U}_{\infty }}{{U}_{w}+{U}_{\infty }},$$

$$\psi =\sqrt{x{\nu }_{\infty }U} f\left(\xi ,\eta \right); \frac{\partial \psi }{\partial x}=-v; \frac{\partial \psi }{\partial y}=u; {f}_{\eta }=F; u= UF,$$

$$G=\frac{ T- {T}_{\infty }}{\Delta T}, \Delta T= {T}_{w}- {T}_{\infty };$$

$$v= -\frac{1}{2}\sqrt{\frac{U{\nu }_{\infty }}{x}}\left[2\xi {f}_{\xi }-\eta F+f\right]$$

are utilized to convert the Eqs. (10)–(11) into non-dimensional form:

14 $$\frac{\partial }{\partial \eta }\left\{N{F}_{\eta }\right\}-\xi {K}_{1}St(F-\epsilon )+{S}_{1}\left(\frac{1}{2}f{F}_{\eta }-\xi \left(F{F}_{\xi }-{f}_{\xi }{F}_{\eta }\right)\right)+\lambda \xi {S}_{2}G=0,$$

15 $$\frac{\partial }{\partial \eta }\left\{\frac{N}{{(Pr)}_{f}}{P}_{5}{G}_{\eta }\right\}+\frac{N}{{(Pr)}_{f}}{R}_{1}{G}_{\eta \eta }+\xi Re Q G+{S}_{3}\left(\frac{1}{2}f{G}_{\eta }+\xi \left({f}_{\xi }{G}_{\eta }-F{G}_{\xi }\right)\right)=0,$$

with

16 $${\left[\begin{array}{c}F\\ G\end{array}\right]}_{\eta =0}= \left[\begin{array}{c}1-\epsilon \\ 1\end{array}\right]; {\left[\genfrac{}{}{0pt}{}{F}{G}\right]}_{\eta ={\eta }_{\infty }}= \left[\begin{array}{c}\epsilon \\ 0\end{array}\right].$$

The non-dimensional parameters buoyancy ($\lambda$), Reynolds number($Re$), Grashof number($Gr$), Stuart number($St$), radiation(${R}_{1}$), heat source($Q$) are defined, respectively, as follows:

$$\lambda =\frac{Gr}{{Re}^{2}};Re= \frac{UL}{{\nu }_{\infty }}; Gr=\frac{g{\beta }_{f}\Delta T{L}^{3}}{{\nu }_{\infty }^{2}}; St=\frac{{\sigma }_{f}{B}_{0}^{2}L}{{\rho }_{f}U}; {R}_{1}= \frac{16 {\sigma }^{*} {T}_{\infty }^{3}}{3{k}^{*}{k}_{f}}; Q=\frac{{Q}_{0}{\nu }_{\infty }}{{{(C}_{p}\rho )}_{f}{U}^{2}}.$$

All the other constants and coefficients are prescribed below:

$${a}_{1}=\frac{{b}_{2}\Delta T}{{b}_{1}+{b}_{2}{T}_{\infty }}; {a}_{2}= {b}_{1}+{b}_{2}{T}_{\infty }; {a}_{3}={a}_{1}{a}_{2}; {d}_{1}= {c}_{1}+{c}_{2}{T}_{\infty }; {d}_{2}={c}_{2}\Delta T,$$

$$N=\frac{1}{1+{a}_{1}G}; {(\text{Pr})}_{f}=\frac{1}{{d}_{1}+{d}_{2}G}; {P}_{5}=\frac{{P}_{1}+{P}_{2}G}{{P}_{3}+{P}_{4}G}; {P}_{6}=\frac{{d}_{2}-{a}_{1}{d}_{1}}{{\left(1+{a}_{1}G\right)}^{2}};$$

$${P}_{1}= {a}_{2}{\Psi }_{s}+2{C}_{p}{d}_{1}-2\phi \left({C}_{p}{d}_{1}-{a}_{2}{k}_{s}\right); {P}_{2}= {a}_{3}{k}_{s}+2{C}_{p}{d}_{2}-2\phi \left({C}_{p}{d}_{2}-{a}_{3}{k}_{s}\right),$$

$${P}_{3}= {a}_{2}{k}_{s}+2{C}_{p}{d}_{1}+\phi \left({C}_{p}{d}_{1}-{a}_{2}{k}_{s}\right); {P}_{4}= {a}_{3}{k}_{s}+2{C}_{p}{d}_{2}+\phi \left({C}_{p}{d}_{2}-{a}_{3}{k}_{s}\right),$$

$${P}_{7}=\frac{{P}_{2}{P}_{3}-{P}_{1}{P}_{4}}{{\left({P}_{3}+{P}_{4}G\right)}^{2}}; {P}_{8}=-{a}_{1}\frac{\left({d}_{2}-{a}_{1}{d}_{1}\right)}{{\left(1+{a}_{1}G\right)}^{3}}; {P}_{9}=-{P}_{4}\frac{\left({P}_{2}{P}_{3}-{P}_{1}{P}_{4}\right)}{{\left({P}_{3}+{P}_{4}G\right)}^{3}},$$

$${S}_{1}=\left\{1-\left(1-\frac{{\left(\rho \right)}_{{s}_{1}}}{{\left(\rho \right)}_{f}}\right){\phi }_{{s}_{1}}-\left(1-\frac{{\left(\rho \right)}_{{s}_{2}}}{{\left(\rho \right)}_{f}}\right){\phi }_{{s}_{2}}\right\}\sqrt{{\left(1-\phi \right)}^{5}},$$

$${S}_{2}=\left\{1-\left(1-\frac{{\left(\rho \beta \right)}_{{s}_{1}}}{{\left(\rho \beta \right)}_{f}}\right){\phi }_{{s}_{1}}-\left(1-\frac{{\left(\rho \beta \right)}_{{s}_{2}}}{{\left(\rho \beta \right)}_{f}}\right){\phi }_{{s}_{2}}\right\}\sqrt{{\left(1-\phi \right)}^{5}},$$

$${S}_{3}=\left\{1-\left(1-\frac{{\left({C}_{p}\rho \right)}_{{s}_{1}}}{{\left({C}_{p}\rho \right)}_{s}}\right){\phi }_{{s}_{1}}-\left(1-\frac{{\left({C}_{p}\rho \right)}_{{s}_{2}}}{{\left({C}_{p}\rho \right)}_{s}}\right){\phi }_{{s}_{2}}\right\},$$

$${K}_{1}=\left\{\frac{{\sigma }_{f}+2{\sigma }_{s}-2\phi \left({\sigma }_{f}-{\sigma }_{s}\right)}{{\sigma }_{f}+2{\sigma }_{s}+\phi \left({\sigma }_{f}-{\sigma }_{s}\right)}\right\}\sqrt{{\left(1-\phi \right)}^{5}}\,\text{ where }\,{\sigma }_{s}= {\sigma }_{{s}_{1}}+{\sigma }_{{s}_{2}}.$$

### Salient gradients

#### Friction (${{\varvec{C}}}_{{{\varvec{f}}}_{{\varvec{x}}}})$

$${C}_{{f}_{x}}=\frac{2{\mu }_{hnf}{\left(\frac{\partial u}{\partial y}\right)}_{y=0}}{{\rho }_{f}{u}_{e}^{{*}^{2}}},$$

$$\therefore \sqrt{Re}{C}_{{f}_{x}}=\frac{2}{\left(1+{a}_{1}\right){(1-\phi )}^{2.5}}\frac{{F}_{\eta }\left(\xi ,0\right)}{\sqrt{\xi }}.$$

#### Heat transfer (${{\varvec{N}}{\varvec{u}}}_{{\varvec{x}}}$)

$${Nu}_{x}= \frac{x {q}_{w}}{{k}_{f}\Delta T} \left[where {q}_{w}=- {k}_{hnf}{\left(\frac{\partial T}{\partial y}\right)}_{y=0}\right],$$

$$\Rightarrow {Nu}_{x}= \frac{x{k}_{hnf}{\left(\frac{\partial T}{\partial y}\right)}_{y=0}}{{k}_{f}\Delta T},$$

$$\therefore \frac{{Nu}_{x}}{\sqrt{Re}}=-\sqrt{\xi }{P}_{5}{G}_{\eta }\left(\xi ,0\right).$$

## Generation of entropy

The EG model for MHD hybrid nanofluid can be written as^53^:

$$S_{{gen}} = \left( {\underbrace {{\frac{1}{{T_{\infty }^{2} }}\left( {k_{{hnf}} \left( {\frac{{\partial T}}{{\partial y}}} \right)^{2} + \frac{{16\sigma ^{*} T_{\infty }^{3} }}{{3k^{*} }}\left( {\frac{{\partial T}}{{\partial y}}} \right)^{2} } \right)}}_{{HTI}}} \right) + \left( {\underbrace {{\frac{{\mu _{{hnf}} }}{{T_{\infty } }}\left( {\frac{{\partial u}}{{\partial y}}} \right)^{2} + \frac{{\sigma _{{hnf}} B_{0}^{2} }}{{T_{\infty } }}}}_{{FFI}}u} \right).$$

The first brace (HTI) includes the terms representing irreversibility for heat transfer, terms inside second brace (FFI) conveys the irreversibility for fluid friction. The characteristics entropy rate ${S}_{0}=\frac{\Delta {T}^{2}{k}_{f}}{{L}^{2}{T}_{\infty }^{2}}$ is utilized to get the dimensionless form (${S}_{G}$) of total entropy (${S}_{gen}$) i.e., ${S}_{G}=\frac{{S}_{gen}}{{S}_{0}}={N}_{1}+{N}_{2}$ where

$${N}_{1}=\frac{HTI}{{S}_{0}}=\frac{1}{\xi }\left[{P}_{5}+R\right]Re {G}_{\eta }^{2},$$

$${N}_{2}=\frac{FFI}{{S}_{0}}=\left(\frac{1}{\sqrt{{\left(1-\phi \right)}^{5}}} \frac{{F}_{\eta }^{2}}{\xi }+{K}_{1}St {F}^{2}\right)\frac{Re Br}{\Omega }.$$

Here the notations $\Omega = \frac{\Delta T}{{T}_{\infty }},$ and $Br=\frac{{U}^{2}{\mu }_{f}}{{k}_{f}\Delta T}$ stand for temperature ratio and Brinkman number, respectively. The comparative study of relative irreversibility sources can be accomplished with Bejan number (Be). Mathematically, it is defined by

$$Be=\frac{HTI}{HTI+FFI}=\frac{{N}_{1}}{{N}_{1}+{N}_{2}}=\frac{\text{Irreversiblity due to heat transfer}}{\text{total local entropy}}.$$

## Numerical method and validation

The set of coupled non-linear Eqs. (14–15) has been made linear by employing the quasilinearization technique and the equations turned into

17 $${E}_{11}^{(k)}{F}_{\eta \eta }^{(k+1)}+{E}_{12}^{(k)}{F}_{\eta }^{(k+1)}+{E}_{13}^{(k)}{F}_{\xi }^{(k+1)}+{E}_{14}^{(k)}{F}^{(k+1)}+{E}_{15}^{(k)}{G}_{\eta }^{(k+1)}+{E}_{16}^{(k)}{G}^{(k+1)}={E}_{17}^{(k)},$$

18 $${E}_{21}^{(k)}{G}_{\eta \eta }^{(k+1)}+{E}_{22}^{(k)}{G}_{\eta }^{(k+1)}+{E}_{23}^{(k)}{G}_{\xi }^{(k+1)}+{E}_{24}^{(k)}{G}^{(k+1)}+{E}_{25}^{(k)}{F}^{(k+1)}={E}_{26}^{(k)},$$

with the boundary constraints

19 $${\left[\genfrac{}{}{0pt}{}{{F}^{\left(k+1\right)}}{{G}^{\left(k+1\right)}}\right]}_{\eta =0}= \left[\genfrac{}{}{0pt}{}{1-\epsilon }{1}\right]; {\left[\genfrac{}{}{0pt}{}{{F}^{\left(k+1\right)}}{{G}^{\left(k+1\right)}}\right]}_{\eta ={\eta }_{\infty }}= \left[\genfrac{}{}{0pt}{}{\epsilon }{0}\right].$$

Here the system (17–18) is linear for iterative indices $\left(k+1\right)$ as superscripts with the coefficients:

$${E}_{11}=N; {E}_{12}=-{a}_{1}{G}_{\eta }{N}^{2}+{S}_{1}\left[f+\xi {f}_{\xi }\right]; {E}_{13}={-S}_{1}\xi F,$$

$${E}_{14}={-\xi S}_{1}{F}_{\xi }-\xi {K}_{1}St; {E}_{15}=-{a}_{1}{F}_{\eta }{N}^{2};$$

$${E}_{16}=-{a}_{1}{F}_{\eta \eta }{N}^{2}+2{a}_{1}^{2}{F}_{\eta }{G}_{\eta }{N}^{3}+\xi \lambda {S}_{2};$$

$${E}_{17}={-{a}_{1}{F}_{\eta \eta }G{N}^{2}-{a}_{1}{F}_{\eta }{G}_{\eta }{N}^{2}+2{a}_{1}^{2}{F}_{\eta }{G}_{\eta }G{N}^{3}-S}_{1}\xi F{F}_{\xi }-\epsilon \xi {K}_{1}St; {E}_{21}=\frac{N}{{(Pr)}_{f}}\left[{P}_{5}+{R}_{1}\right],$$

$${E}_{22}=2{G}_{\eta }\left[{P}_{5}{P}_{6}+\frac{N}{{(Pr)}_{f}}{P}_{7}\right]+{S}_{3}\left(\frac{1}{2}f+\xi {f}_{\xi }\right); {E}_{23}={-S}_{2}\xi F,$$

$${E}_{24}={G}_{\eta \eta }\left[{P}_{6}\left({P}_{5}+{R}_{1}\right)+\frac{N}{{(Pr)}_{f}}{P}_{7}\right]+{G}_{\eta }^{2}\left[2{P}_{6}{P}_{7}+{P}_{5}{P}_{8}+\frac{N}{{(Pr)}_{f}}{P}_{9}\right]+\xi Q;$$

$${E}_{25}={-S}_{2}\xi {G}_{\xi };$$

$${E}_{26}={GG}_{\eta \eta }\left[\left({P}_{5}+{R}_{1}\right){P}_{6}+\frac{N}{{(Pr)}_{f}}{P}_{7}\right]+{G}_{\eta }^{2}\left[{P}_{5}{P}_{6}+\frac{N}{{(Pr)}_{f}}{P}_{7}\right]{-S}_{2}\xi F{G}_{\xi }+G{G}_{\eta }^{2}\left[{2P}_{6}{P}_{7}+{P}_{5}{P}_{8}+\frac{N}{{(Pr)}_{f}}{P}_{9}\right];$$

At this point, the following finite difference (implicit) schemes

$${F}_{\eta \eta }=\frac{\left({F}_{m, n-1}-2{F}_{m, n}+{F}_{m, n+1}\right)}{{\left(h\right)}^{2}},$$

$${F}_{\eta }=\frac{\left({F}_{m, n+1}-{F}_{m, n-1}\right)}{2h},$$

$${F}_{\xi }=\frac{\left({F}_{m, n}-{F}_{m-1, n}\right)}{k},$$

transform Eqs. (17–18) into a set of algebraic equations as:

20 $${A}_{n}{W}_{m,n-1}+{B}_{n}{W}_{m,n}+{C}_{n}{W}_{m,n+1}={D}_{n} ; \left(2\le n\le \overline{N }\right),$$

for fixed m, where $\overline{N }$ is the number intervals of this mesh system and the vectors, coefficient matrices are:

$${W}_{m,n}={\left[\begin{array}{c}F\\ G\end{array}\right]}_{m, n};{D}_{n}={\left[\begin{array}{c}{d}_{1}\\ {d}_{2}\end{array}\right]}_{n}; {A}_{n}={\left[\begin{array}{cc}{a}_{11}& {a}_{12}\\ {a}_{21}& {a}_{22}\end{array}\right]}_{n};$$

$${B}_{n}={\left[\begin{array}{cc}{b}_{11}& {b}_{12}\\ {b}_{21}& {b}_{22}\end{array}\right]}_{n};{C}_{n}={\left[\begin{array}{cc}{c}_{11}& {c}_{12}\\ {c}_{21}& {c}_{22}\end{array}\right]}_{n},$$

where the entries of ${A}_{n}$, ${B}_{n}$, ${C}_{n}$, and ${D}_{n}$ are defined as:

$${a}_{11}={E}_{11}-\frac{h}{2}{E}_{12}, {a}_{12}=-\frac{h}{2}{E}_{12},$$

$${a}_{21}=0, {a}_{22}={E}_{21}-\frac{h}{2}{E}_{22},$$

$${b}_{11}=-2{E}_{11}+\frac{{h}^{2}}{k}{E}_{13}+{h}^{2}{E}_{14}, {b}_{12}={h}^{2}{E}_{16},$$

$${b}_{21}={h}^{2}{E}_{25}, {b}_{22}=-2{E}_{21}+\frac{{h}^{2}}{k}{E}_{23}+{h}^{2}{E}_{24},$$

$${c}_{11}={E}_{11}+\frac{h}{2}{E}_{12}, {c}_{12}=\frac{h}{2}{E}_{15},$$

$${c}_{21}=0, {c}_{22}={E}_{21}+\frac{h}{2}{E}_{22},$$

$${d}_{1}={h}^{2}{E}_{17}+\frac{{h}^{2}}{k}{E}_{13}{F}_{m-1, n}, {d}_{2}={h}^{2}{E}_{26}+\frac{{h}^{2}}{k}{E}_{23}{G}_{m-1, n},$$

${W}_{1}$ and ${W}_{\overline{N }+1}$ at the boundaries (at $\eta =0$ and $\eta ={\eta }_{\infty }$) become:

21 $${W}_{1}={\left[\begin{array}{c}F\\ G\end{array}\right]}_{m,\eta =0}=\left[\begin{array}{c}1-\epsilon \\ 1\end{array}\right] ; {W}_{\overline{N }+1}={\left[\begin{array}{c}F\\ G\end{array}\right]}_{m,\eta ={\eta }_{\infty }}=\left[\begin{array}{c}\epsilon \\ 0\end{array}\right].$$

Hereafter, Varga’s algorithm^34^, as defined below, is used to solve Eqs. (20) with constraints given by Eq. (21).

$${W}_{n}=-{E}_{n}{W}_{n+1}+{J}_{n} , 1\le n\le \overline{N },$$

where $${E}_{n}={\left\{{B}_{n}-{A}_{n}{E}_{n-1}\right\}}^{-1}{C}_{n} ;$$

$${J}_{n}={\left\{{B}_{n}-{A}_{n}{E}_{n-1}\right\}}^{-1}\left\{{D}_{n}-{A}_{n}{J}_{n-1}\right\};2\le n\le \overline{N },$$

$${E}_{1}={E}_{\overline{N }+1}=\left[\begin{array}{cc}0& 0\\ 0& 0\end{array}\right], {J}_{1}=\left[\begin{array}{c}1-\epsilon \\ 1\end{array}\right], {J}_{\overline{N }+1}=\left[\begin{array}{c}\epsilon \\ 0\end{array}\right].$$

The numerical solutions are reached under the strict convergence criterion

$$\left(Max\left\{\left|{\left({F}_{\eta }\right)}_{w}^{k+1}-{\left({F}_{\eta }\right)}_{w}^{k}\right|,\left|{\left({G}_{\eta }\right)}_{w}^{k+1}-{\left({G}_{\eta }\right)}_{w}^{k}\right|\right\}<{10}^{-4}\right),$$

and compared in Table 4 with previously published works^54–56^ and found in a friendly match-up (see Table 4).

**Table 4:** Comparison of current results with available works^54–56^ in literature for the case of steady-state with $\epsilon =0, \phi =0, \gamma =0, \lambda =0, {B}_{0}=0, {q}_{r}=0, {Q}_{0}=0$ for $-{G}_{\eta }(0)$ at $\eta =0$.

$Pr$	2	5	7	10	100
Soundalgekar and Murty^54^	$0.6831$	–	–	$1.6808$	–
Chen^55^	$0.68324$	–	$1.38619$	$1.68008$	$5.54450$
Singh et al.^56^	$0.6830$	$1.151$	$1.386$	$1.6801$	$5.5450$
Present results	$0.6831$	$1.1512$	$1.3861$	$1.6801$	$5.5448$

## Results and discussion

The investigation of bi-convective MHD flow in light of temperature-sensorial water properties with radiation, thermal suction/injection effects is accomplished in this manuscript considering $Cu+{Al}_{2}{O}_{3}/\text{water}$ hybrid nanofluid as working fluid. The acquired outcomes are featured out graphically to analyse the flow features, transport characteristics and energy distribution in comprehensive approach.

### Velocity

**Figure 2:**
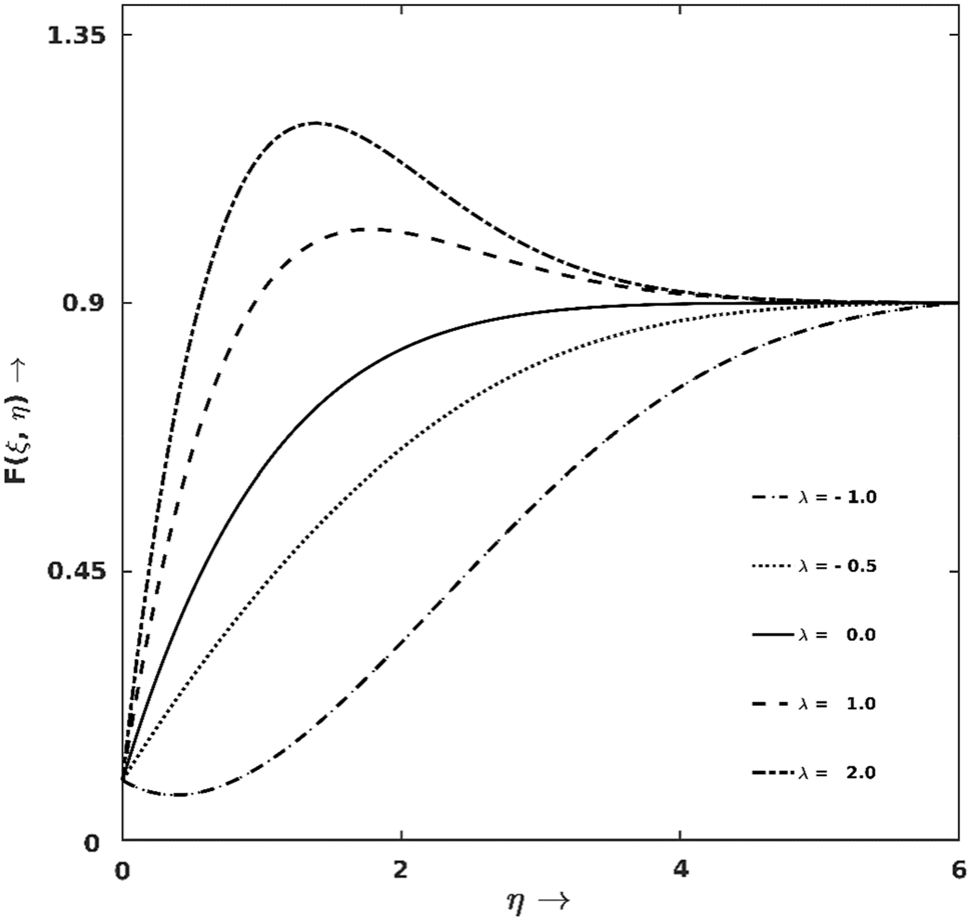
$$\lambda$$ effect on $$F$$.

Figure 2 is plotted to display the variable behaviour of the flow intensity ($F(\xi ,\eta )$) against the buoyancy force $\lambda$. It may be noted that $F(\xi ,\eta )$ increases with $\lambda$, sometimes overshoot occurs. In the physical aspect, assisting buoyancy force always surpluses pressure gradient in flow and enhances flow intensity. As numerical supporting evidence, it is seen for $\lambda =1$ and $\lambda = 2$ at $\xi =0.5, \eta =1.40$ that the velocity overshoots are $15\%$ and $33\%$, respectively. In contrast, $F(\xi ,\eta )$ decreases for $\lambda <0$, and in this case, for $\lambda = -1.0$ backflow is recorded within the region $0.0<\eta \le 0.85, \xi =0.5$.

### Temperature

**Figure 3:**
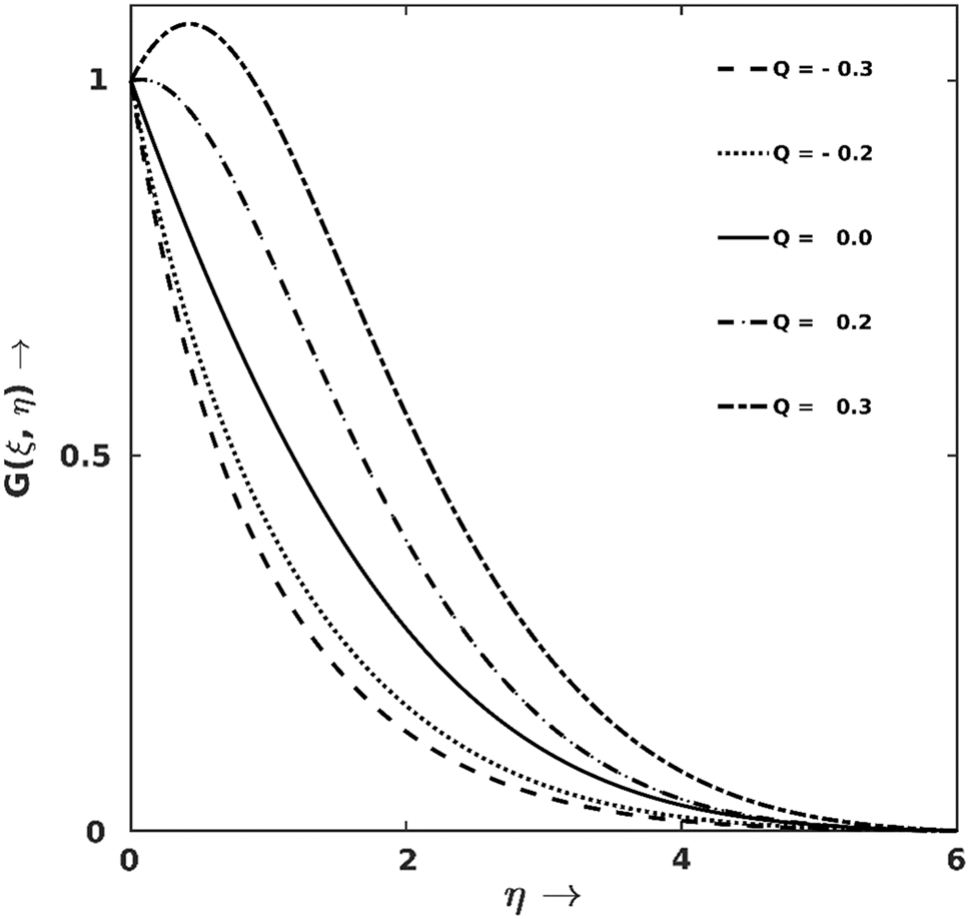
$$Q$$ effect on $$G$$.

The changes in temperature-profile $(G(\xi ,\eta ))$ against the variations of thermal sink/source $(Q)$ is elaborated graphically in Fig. 3. Since the heat source ($Q>0$) is kept in the BL to enhance heat energy, $G(\xi ,\eta )$-enhancement concerning $Q>0$ is unambiguous. Specifically, at $\xi =0.45, \eta =0.5$, varying $Q$ from $0.0$ to $0.3$ and $0.0$ to $-0.3,$ $G(\xi ,\eta )$-profile increases and decreases, respectively, by $8\%$ and $39\%$.

### Gradients

#### Skin friction

**Figure 4:**
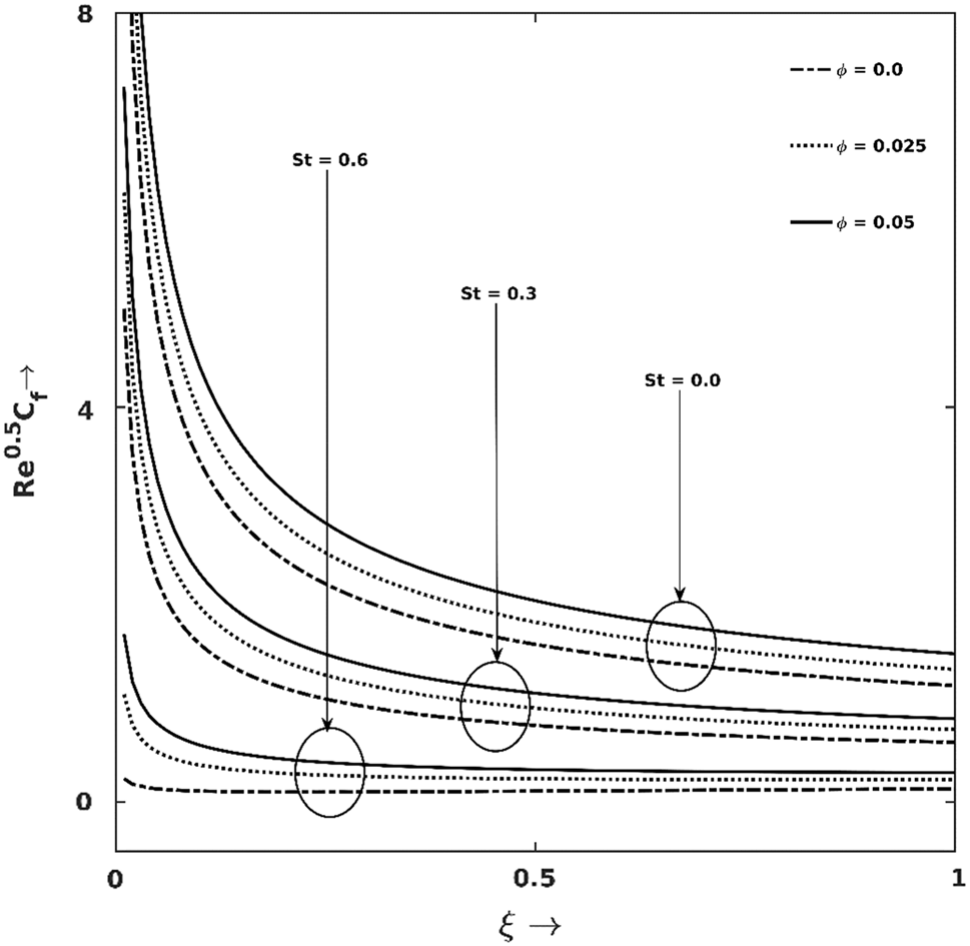
Friction coefficient ($\sqrt{Re}{C}_{{f}_{x}}$) graph for different $St$ and $\phi$.

The variation characteristics of friction coefficient ($\sqrt{Re}{C}_{fx}$) against different magnitudes of $St$ and $\phi$ are demonstrated in Fig. 4, which reflects that $\sqrt{Re}{C}_{fx}$ is a decreasing function of $St$ but an increasing function of $\phi$. The Lorentz’s force associated with $St$ is active to detract the BL region's flow intensity, and thus friction gets dissipated. On the other hand, enhancement of tiny nanoparticles in the fluid causes richer mass density and thus increases hybrid nanofluid’s friction forces and finally, $\sqrt{Re}{C}_{fx}$ increases. At the instant $\xi =0.5$ with $\phi =0.025$ enhancing $St$ of strengths $0.3$ and $0.6$ from $0$, $\sqrt{Re}{C}_{fx}$ reduces by $48\%$ and $87\%$, respectively.

#### Nusselt number

**Figure 5:**
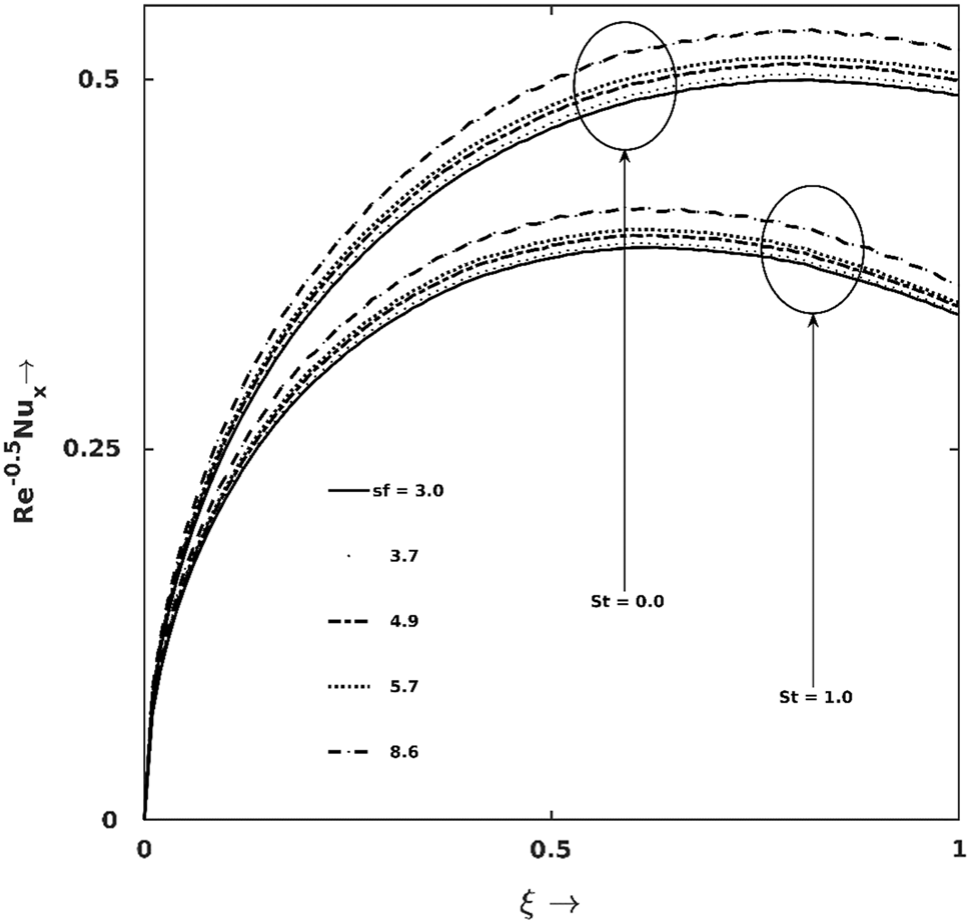
Nusselt number $\left(\frac{{Nu}_{x}}{\sqrt{Re}}\right)$ graph for different $St$ and nanoparticles’ shape factor ($sf$).

The corresponding impact of $St$ on thermal transport performance ($\frac{{Nu}_{x}}{\sqrt{Re}}$) in combination with nanoparticles’ shape effects are portrayed in Fig. 5. The results indicate that the outlying force field ($St$) has a destructive impact on $\frac{{Nu}_{x}}{\sqrt{Re}}$, and among all the considered shapes, spherical-shaped nanoparticles affect most. In particular at $\xi =0.5,$ the decrement in sphericity $\Omega$ (i.e., increment in $sf=\frac{3}{\Omega }$) from $1.0$ to $0.36$ enhances $\frac{{Nu}_{x}}{\sqrt{Re}}$ almost by $7\%$.

**Figure 6:**
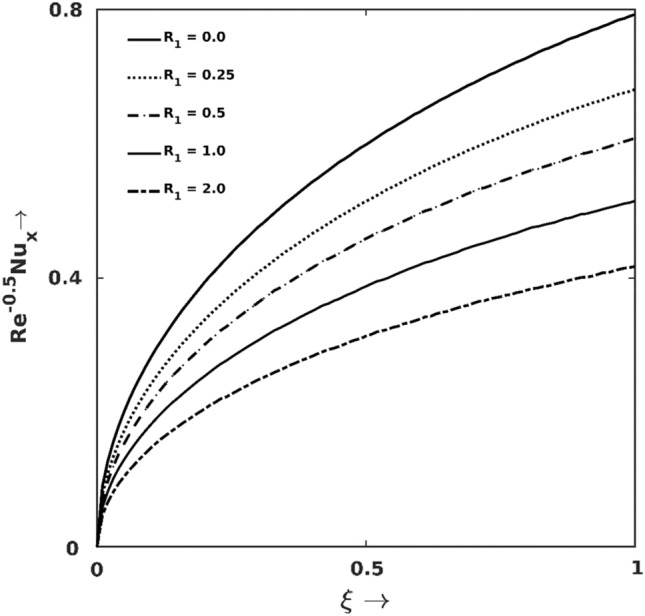
Nusselt number $\left(\frac{{Nu}_{x}}{\sqrt{Re}}\right)$ graph for different ${R}_{1}$.

Figure 6 depicts the effects of thermal radiation (${R}_{1}$) on local thermal transport coefficient ($\frac{{Nu}_{x}}{\sqrt{Re}}$) and it is clearly visible in the graph that $\frac{{Nu}_{x}}{\sqrt{Re}}$ is a decreasing function of ${R}_{1}$. Basically, the increasing magnitude of ${R}_{1}$ directly enhances systems’ temperature, and the fluid in BL tries to become thermally equipoise. Hence temperature gradient gets reduced, which results in less thermal transport. At the instant $\xi =1.0$, reduction in $\frac{{Nu}_{x}}{\sqrt{Re}}$ is $35\%$ for imposing ${R}_{1}$ of strength $1.0$.

### Entropy production and Bejan lines

**Figure 7:**
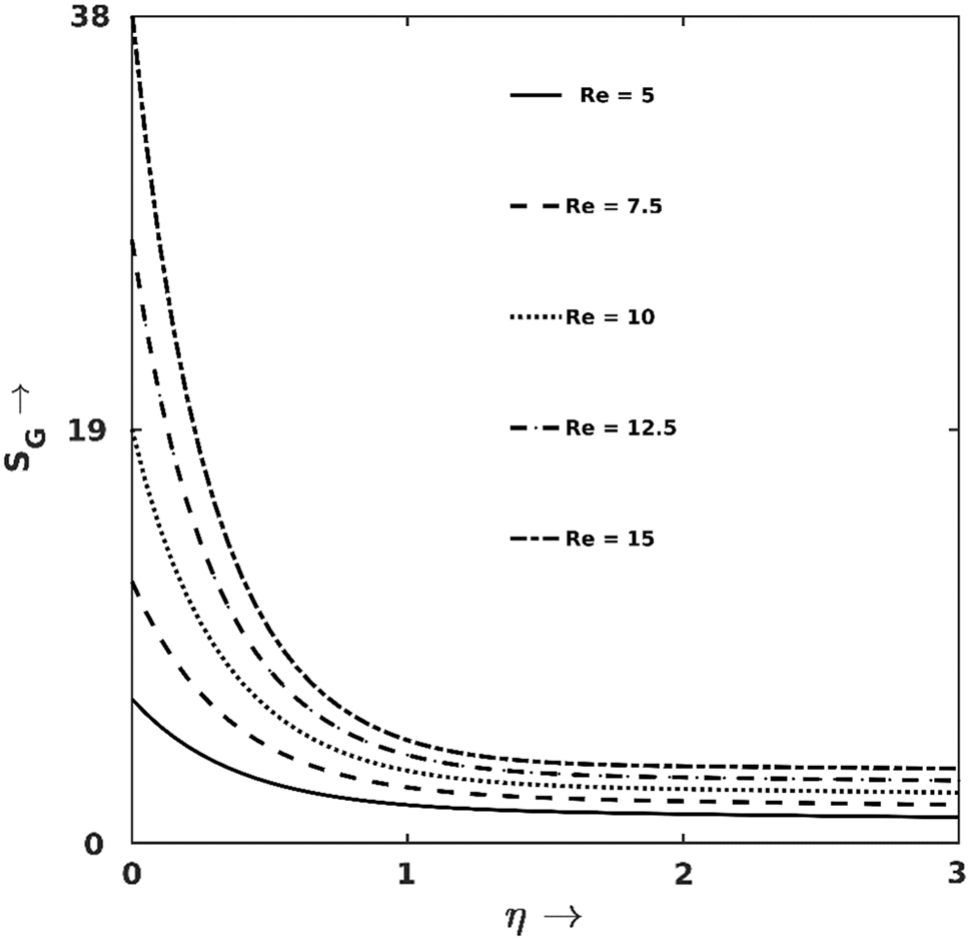
$$Re$$ effect on $${S}_{G}$$.

**Figure 8:**
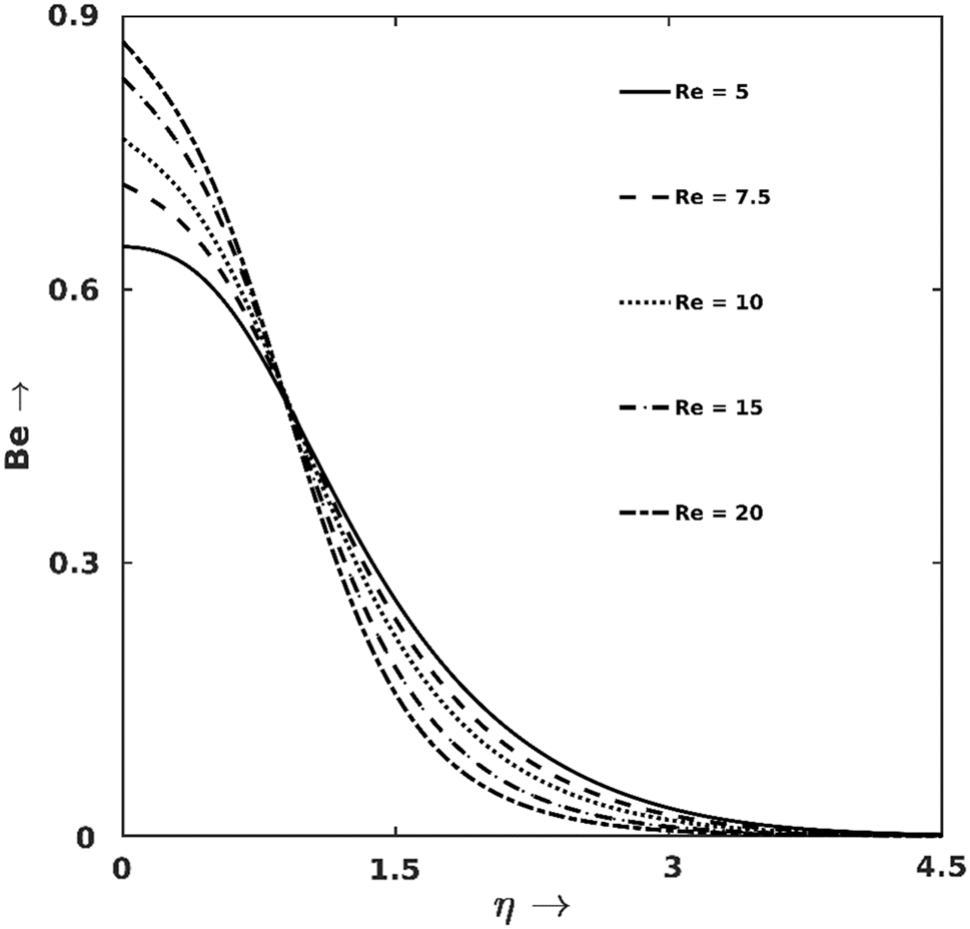
$$Re$$ effect on $$Be$$.

**Figure 9:**
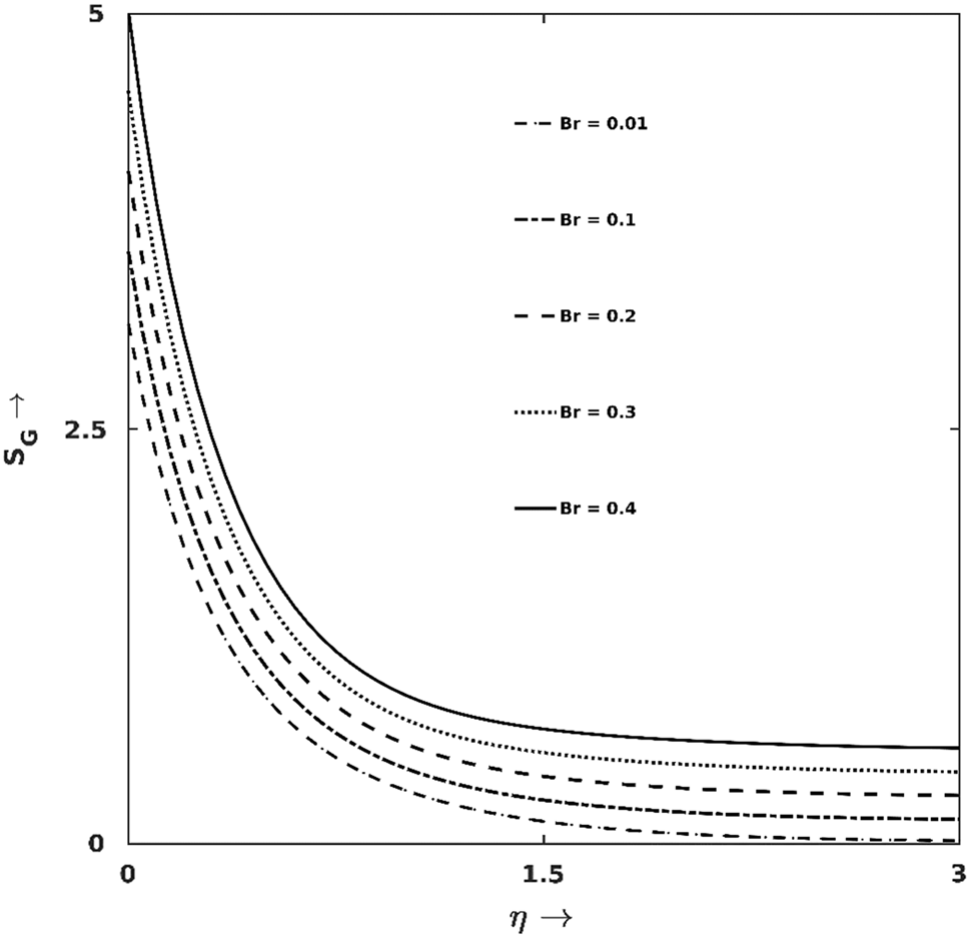
$$Br$$ effect on $${S}_{G}$$.

**Figure 10:**
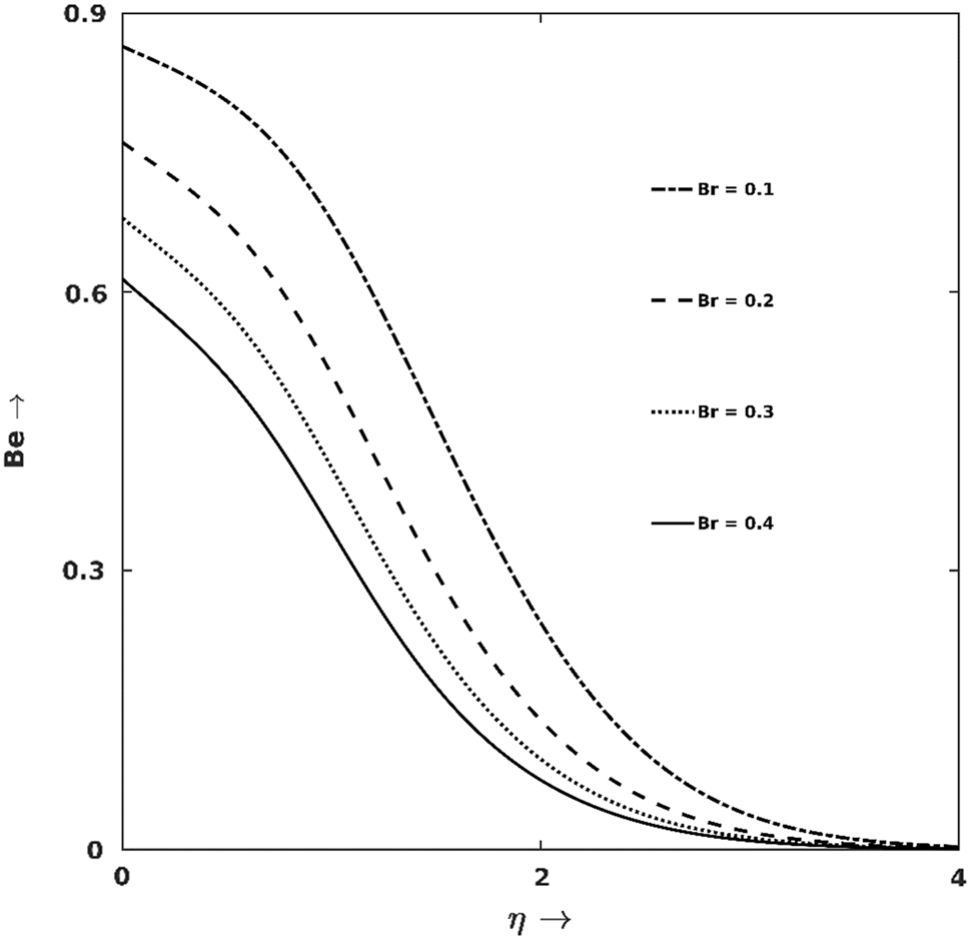
$$Br$$ effect on $$Be$$.

**Figure 11:**
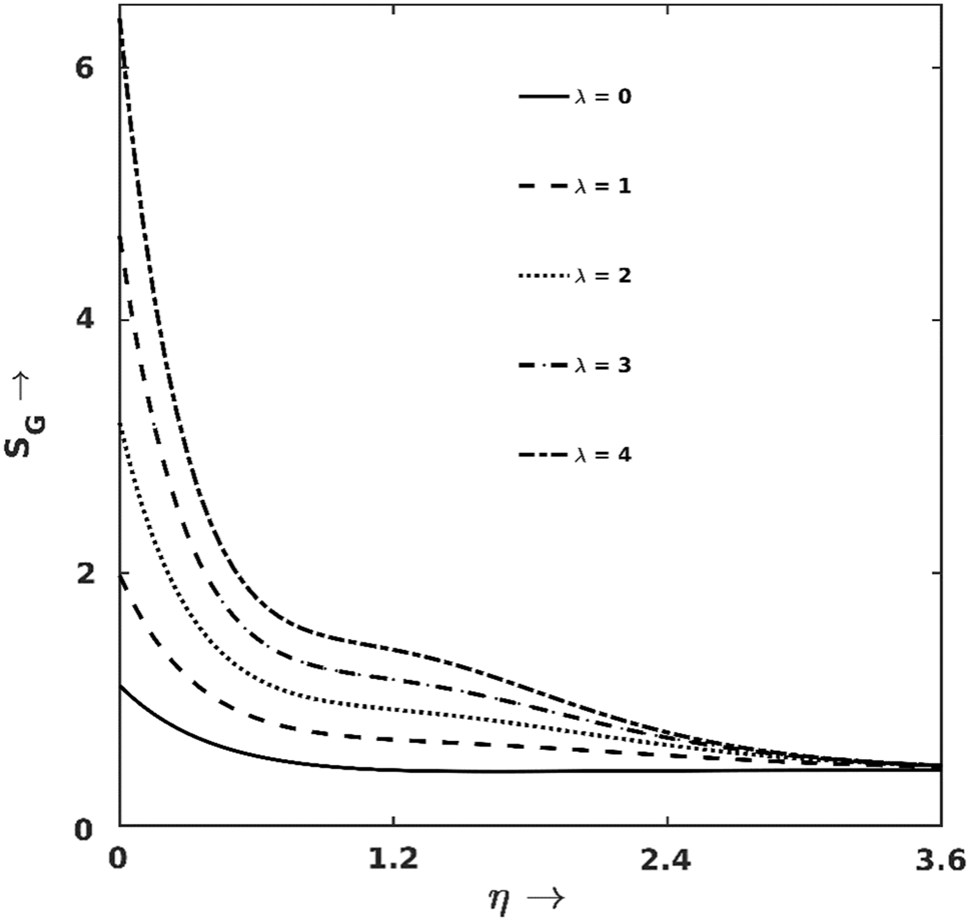
$$\lambda$$ effect on $${S}_{G}$$.

**Figure 12:**
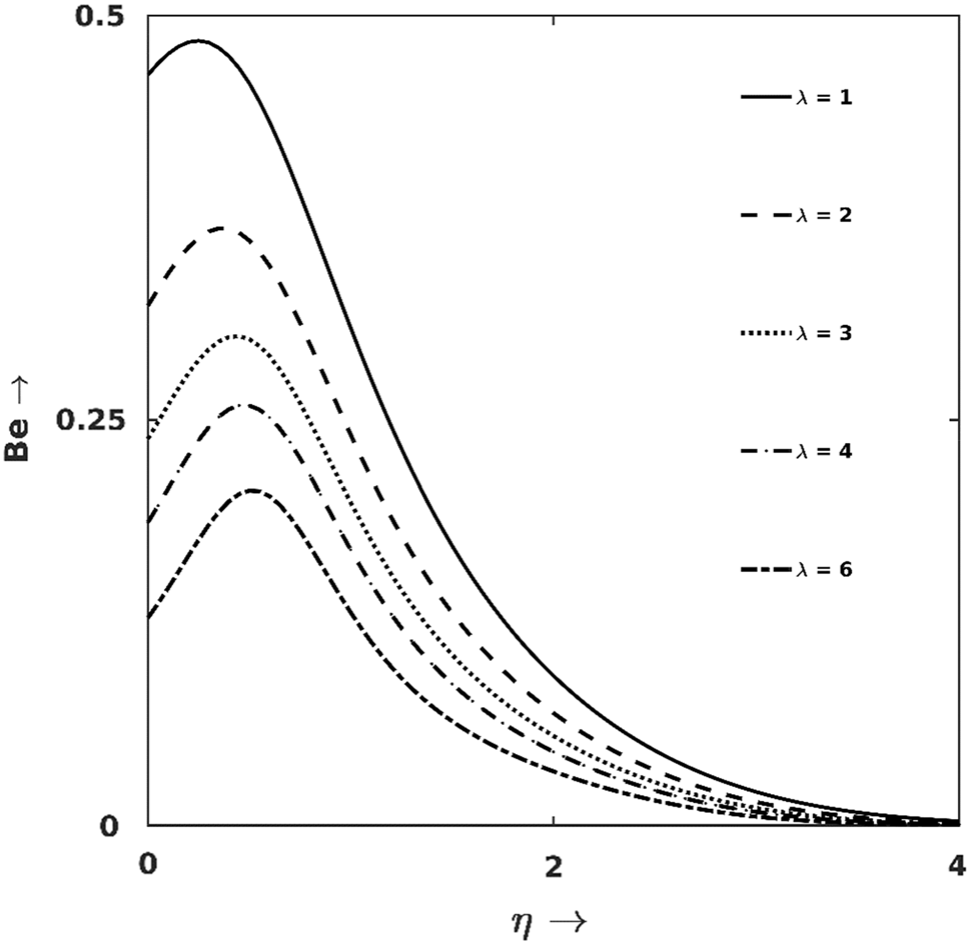
$$\lambda$$ effect on $${S}_{G}$$.

Figures 7, 8, 9, 10, 11 and 12 illuminate the contributions of different salient parameters on the productions of irreversible heats (entropy production ${S}_{G}$) and their respective shares on gross entropy. Figure 7 indicates that the rate of ${S}_{G}$-production increases with $Re$, but $Re$'s contribution on ${S}_{G}$ is immensely high at the surface proximity. Physically, augmentation of $\text{Re}$ increases the entropy generation ${\text{S}}_{\text{G}}$ due to fluid friction and heat transport (via inertia). For higher $Re$, fluid inertia augments thermal transport, i.e., $HTI$ takes over the other irreversibility sources. In contrast for lower $Re$, as viscous force is high, $FFI$ dominates the total ${\text{S}}_{\text{G}}$ close to the wall. Thus, the friction force gets mitigated within the boundary layer and $HTI$ takes over the dominant place. Hence, Bejan lines for lower $Re$ intersect the lines for higher $Re$ within the boundary layer.

Moreover, all the Bejan lines converge to zero at the boundary layer edge since $HTI$ gradually reduces to zero at the edge of the boundary layer. It is also noticed that the surface plays a high intense ${S}_{G}$-production source and is evidenced by the following specific calculation: at $\eta =0.0$, ${S}_{G}$ elevates by $46\%$ for varying $Re$ in $10-12.5$ while the change is only $20\%$ at $\eta =1.5$ for the same variation of $Re$.

Figures 9 and 10 manifested the ${S}_{G}$-production and Bejan line regarding different magnitudes of viscous heating ($Br$). As exhibited in Fig. 12, higher $Br$ boosts ${S}_{G}$ at the wall’s proximity but discloses an opposite trend away from the surface. Lifting up the $Br$ value causes added viscous force to the fluid and enhances frictional heating. This frictional heating turns up excessive ${S}_{G}$-production. This fact is also evidenced in Fig. 10, which shows lifted down Bejan lines for higher $Br$, which physically represents that most ${S}_{G}$-productions are due to frictional heating (FFI), the associated entropy produced in other modes (i.e., HTI and DI) are comparatively less. Analysing the result data, $32\%$ enhancement in ${S}_{G}$ is noticed for changing $Br$ from $0.01$ to $0.2$ at $\eta =0.5$.

Figures 11 and 12 demonstrate how ${S}_{G}$ and $Be$ get affected under the forces of buoyancy ($\lambda$). As one can point out from Fig. 11 that ${S}_{G}$ shows a growing trend for the increase of $\lambda$. The earlier discussions proclaimed that larger $\lambda$ pushes the fluids to move faster generates excessive friction at the wall and hence the irreversibility enhances (via FFI, as shown in Fig. 12). Since the buoyancy effect is induced by the thermal imbalance between the wall and neighbouring fluids, the effect of $\lambda$ is predominantly noticeable at the wall proximity. Hence, the irreversibilities due to $\uplambda$ variation vanish at the boundary layer edge and all ${S}_{G}$-lines converge at the edge of the boundary layer.

## Conclusions

This paper performs an analysis on a hybrid nano-liquid flow for an inclined surface under various realistic and practical physical situations by considering the basic temperature-sensorial inheriting characteristics (thermos-physical) of base fluid water. The bearings of flow features, thermal transport characteristics, and EG of magnetized bi-convective hybrid nano-liquid flow with nanoparticles’ sphericity, radiation and thermal source/sink effects are studied in this investigation. The immensely nonlinear PDEs are changed into suitable form and then into linear form utilizing compatible transformation and quasilinearization techniques, respectively. Hereafter, implicit difference methods changed the resulting equations into a matrix system which was further solved by Vargas’ block matrix iterative method. The acquired results of this study are manifested in graphs and discussed in details. The concluding remarks from the investigated results are summarized and expressed with numerical percentile calculations as observed in this specific study:

i. The trend of $F(\xi ,\eta )$-profiles shows increment for assisting ($\lambda >0$) and decrement for opposing buoyancy ($\lambda <0$). In particular, for $\lambda =2$, almost $33\text{\%}$ overshoot is observed when at $\eta =1.40, \xi =0.5$ but in contradiction almost $25\text{\%}$ backflow is noticed at $\eta =0.4, \xi =0.5$ when $\lambda =-1.0$.
ii. Temperature-profile ($G(\xi ,\eta )$) rising along with the heat source strength $Q$.
iii. Significant reduction in friction is happened under the effect of MHD parameter $St$. In particular, at $\xi =1.0$, imposing $St$ of magnitude $0.6$ on $\sqrt{Re}{C}_{fx}$ deduces it almost by $87\text{\%}$.
iv. Friction $\left(\sqrt{Re}{C}_{{f}_{x}}\right)$ escalates for increasing the amount of nanoparticles, specifically, $\sqrt{Re}{C}_{fx}$ enhances approximately by $40\text{\%}$ for increasing $\phi$ from $0.0$ to $0.05$.
v. Thermal transport coefficient mitigates under the effect of MHD parameter $St$. Particularly, at $\xi =1.0$, imposing $St$ of magnitude $1.0$ on $\frac{{Nu}_{x}}{\sqrt{Re}}$ deduces it almost $30\text{\%}$.
vi. The heat transport is enhanced by $7\text{\%}$ as the nanoparticles’ sphericity ($\Omega =\frac{3}{sf}$) goes down from $\Omega =1$ to $\Omega =0.36$*.*
vii. The thermal transport rate $\frac{{Nu}_{x}}{\sqrt{Re}}$ is drastically affected by radiation $\left({R}_{1}\right)$. Numerical enumeration on $\frac{{Nu}_{x}}{\sqrt{Re}}$ at $\xi =1.0$ exposes $35\text{\%}$ redution for applying ${R}_{1}$ of strength $1.0$.
viii. The rate of entropy production $\left({S}_{G}\right)$ is cumulative for enhancing estimations of $Re, Br$ and $\lambda$.
ix. Irreversibility owing to frictional heating (*FFI*) takes the dominant place over the other sources (*HTI, DI)* as $Br$ and $\lambda$ increases.
x. Irreversibility due to *HTI* plays the major role in ${S}_{G}$-production over other sources (*FFI, DI*) for higher $Re$ and lower magnitudes of $Br$ and $\lambda$.
xi. The bounding surface acts as a strong source of ${S}_{G}$-production.
xii. The enhancing variation in ${S}_{G}$ is $58\%$ for changing $\lambda$ in the range $1.0-4.0$.

## Data Availability

All data generated or analyzed during this study are included in this published article. Also, the datasets used and/or analyzed during the current study are available from the corresponding author on reasonable request.

## List of symbols

${B}_{0}$: Outlying magnetic field
${C}_{p}$: Specific heat capacitance
g: Gravity
k: Conductivity (thermal)
L: Reference length
${q}_{r}$: Radiation heat flux
${Q}_{0}$: Heat sink/source
$\beta$: Coefficient of volumetric expansion (thermal)
$$\epsilon$$: Velocity ratio
$$\rho$$: Density
$$\sigma$$: Conductivity (electrical)
$\phi$: Nanoparticle volume percentage
$$\psi$$: Streamfunction

## Abbreviations

EG: Entropy generation
BL: Boundary layer
HTI: Heat transfer irreversibility
FFI: Fluid friction irreversibility

## Nondimentional functions

f, F: Streamfunction and velocity, respectively
G: Temperature

## Subscripts

e: Condition at BL edge
w, $\infty$: Conditions at surface and outside of the BL edge, respectively
$$hnf$$: Hybrid nanofluid
$$f$$: Fluid
$s$: Solid particles (nano)
$sf$: Nano-sized particles’ shape factor
$${s}_{1},{s}_{2}$$: $$Cu$$ And $${Al}_{2}{O}_{3}$$ nanoparticles
